# Leukemia multiclass assessment and classification from Microarray and RNA-seq technologies integration at gene expression level

**DOI:** 10.1371/journal.pone.0212127

**Published:** 2019-02-12

**Authors:** Daniel Castillo, Juan Manuel Galvez, Luis J. Herrera, Fernando Rojas, Olga Valenzuela, Octavio Caba, Jose Prados, Ignacio Rojas

**Affiliations:** 1 Department of Computer Architecture and Computer Technology, University of Granada, Granada, Spain; 2 Department of Applied Mathematics, University of Granada, Granada, Spain; 3 Institute of Biopathology and Regenerative Medicine (IBIMER), Center of Biomedical Research (CIBM), University of Granada, Granada, Spain; Instituto Nacional de Medicina Genomica, MEXICO

## Abstract

In more recent years, a significant increase in the number of available biological experiments has taken place due to the widespread use of massive sequencing data. Furthermore, the continuous developments in the machine learning and in the high performance computing areas, are allowing a faster and more efficient analysis and processing of this type of data. However, biological information about a certain disease is normally widespread due to the use of different sequencing technologies and different manufacturers, in different experiments along the years around the world. Thus, nowadays it is of paramount importance to attain a correct integration of biologically-related data in order to achieve genuine benefits from them. For this purpose, this work presents an integration of multiple Microarray and RNA-seq platforms, which has led to the design of a multiclass study by collecting samples from the main four types of leukemia, quantified at gene expression. Subsequently, in order to find a set of differentially expressed genes with the highest discernment capability among different types of leukemia, an innovative parameter referred to as *coverage* is presented here. This parameter allows assessing the number of different pathologies that a certain gen is able to discern. It has been evaluated together with other widely known parameters under assessment of an ANOVA statistical test which corroborated its filtering power when the identified genes are subjected to a machine learning process at multiclass level. The optimal tuning of gene extraction evaluated parameters by means of this statistical test led to the selection of 42 highly relevant expressed genes. By the use of minimum-Redundancy Maximum-Relevance (mRMR) feature selection algorithm, these genes were reordered and assessed under the operation of four different classification techniques. Outstanding results were achieved by taking exclusively the first ten genes of the ranking into consideration. Finally, specific literature was consulted on this last subset of genes, revealing the occurrence of practically all of them with biological processes related to leukemia. At sight of these results, this study underlines the relevance of considering a new parameter which facilitates the identification of highly valid expressed genes for simultaneously discerning multiple types of leukemia.

## Introduction

Cancer is one of the worst diseases to the point of being the leading cause of death worldwide, just behind cardiovascular disease. There are many types of cancer, and each of these may have a gene signature allowing to discern healthy people from people suffering from cancer. In this paper, from the use of transcriptomic data, different types of leukemia will be studied in order to find a leukemia gene signature for each of them. Leukemia, together with Lymphoma and Myeloma, is one of the three different existing blood cancer forms. People that suffer from leukemia produce an abnormal number of immature white blood cells, which collapse the bone marrow and inhibit the creation of the rest of vital blood cells for a balanced immune system and healthy blood. There are two main types of leukemia, being each divided into two subtypes:

- Acute Leukemia appears suddenly and progresses quickly so the treatment has to be urgent.
  - Acute myeloid leukemia (AML): is the most common leukemia in people around 70 years but it has impact in all ages. This malignancy is a heterogeneous group of neoplastic disorders, that are characterized by the proliferation and accumulation of immature hematopoietic cells in the bone marrow and blood. Different genetic factors have been identified that predispose to the development of AML. In this context, the germline predisposition and the existence of haematological disorders antecedent, have been associated with an increased risk of AML [1, 2].
  - Acute lymphoblastic leukemia (ALL): is the most common leukemia in children. About half the cases are in adults and half in children. ALL is also a very heterogeneous disease, characterized by impaired differentiation and proliferation of immature lymphoid cells in the bone marrow and peripheral blood. However, the prognosis of these patients has improved in the last years, especially in children, leading to cure rates approaching 80% to 90% due to the intensification of treatment, patient stratification based on clinical risk factors, and minimal residual disease (MRD) monitoring [3, 4].
- Chronic Leukemia: symptoms appears more slowly, maybe in months or even years.
  - Chronic myeloid leukemia (CML): it is very unusual and affects only 700 people per year. CML, defined as a clonal myeloproliferative disorder, was the first human malignancy associated to a consistent chromosomal abnormality and is characterized by the presence of the fusion oncogene BCR-ABL. Clinical symptoms associated to this disease include hypercellular bone marrow, anemia, platelet dysfunction, and an increase in the number of leukocytes, especially neutrophils and immature myeloid cells [5, 6].
  - Chronic lymphocytic leukemia (CLL): it is more common in people over 60 years and is very rare in people under 40 years. CLL is a common B-cell tumor, characterized by the gradual accumulation of clonally expanded CD5+ B lymphocytes in peripheral lymphoid organs, secondary lymphoid organs, and bone marrow. It is also a genetic and biological complex disease, and the most commonly used factors to stratify CLL patients are the mutational status of the variable portion of the immunoglobulin gene, the deletion of the chromosome 17p and TP53 gene mutations [7, 8].

In this paper, transcriptomic data will be used in order to find biomarkers for each of the types of leukemia mentioned before. For that, two gene quantification technologies (Microarray and RNA-seq) will be utilized to obtain gene expression signature. Both technologies are briefly described next.

### Microarray technology

Microarray technology has been the most widely used gene quantification technology in the last two decades, until the arrival of Next Generation Sequencing (NGS) techniques. Even nowadays, Microarray is still in use due to the low cost of this technology in comparison with RNA-seq. Microarray was the first technology that managed to measure simultaneously the expression levels of all genes. Furthermore, a lot of Microarray platforms or manufacturers can be taken into account. The most extended Microarray platforms are Affymetrix and Illumina [9, 10]. Nevertheless, there are other very important Microarray manufacturers such as Agilent, Exiqon or Taqman. The expression values are obtained by means of microscopic DNA spots attached to a solid surface which has followed a hybridization process. Once this process is completed, a laser is used to measure the gene expression values [11–13]. Finally, the quantification levels are calculated and exported to a .CEL file [14].

### RNA-seq technology

Although Microarray has been the best gene quantification technology since the ninety decade, RNA-seq was consolidated as the most powerful and newest technology since the last decade. As a natural evolutionary step in the gene quantification technologies, RNA-seq is gradually replacing the widespread use of Microarray. There exist many manufacturers that work with RNA-seq but, nowadays Illumina leads the RNA-seq sequencing technology market. Although its application was originally intended for genomic transcription study, it also allows achieving a mapping between the levels of transcription and gene expression [15]. Thanks to this, it is possible to combine gene expression levels from both Microarray and RNA-seq. This is achieved through the quantification of the total number of reads that are mapped to each locus in the transcriptome assembly step. RNA-seq has many advantages in comparison with Microarray, which are explained below.

### Comparison between both technologies

RNA-seq offers an important number of advantages over Microarrays, although the cost of RNA-seq experiments is nowadays still higher than Microarray technology’s:

- RNA-seq allows detecting the variation of a single nucleotide.
- RNA-seq does not require genomic sequence knowledgement.
- RNA-seq provides quantitative expression levels.
- RNA-seq provides isoform-level expression measurements.
- RNA-seq offers a broader dynamic range than Microarrays.

All these advantages of RNA-seq in comparison with Microarray were clear described by Wang et al. [15]. There are many significant Microarray experiments already available for the research community and, there is also even a higher number of Microarray datasets that have not been analyzed so far. These datasets might have information that could reveal important facts and candidate biomarkers. In any case, there is no doubt that RNA-seq is the present technology, but it can also take advantage of the available data from Microarray technology. As Nookaew et al. explained, there is a high consistency between RNA-seq and Microarray, which encourages to continue using Microarray as a versatile tool for gene expression analysis [16]. Furthermore, in our previous paper, an integration with breast cancer data using both Microarray and RNA-seq technologies was performed, achieving results that support the integration of these technologies [17].

In this research, a novel pipeline has been addressed with the purpose of taking advantage of both RNA-seq and Microarray gene quantification technologies. Furthermore, this integration has been done at multiclass level, due to the nature of the problem studied. At sight of this novel analysis, a new parameter has been introduced for extracting a set of candidate signature biomarkers, this parameter will be called COVERAGE or COV from now on and it will be used together with the Log-Fold Change (LFC) in order to find differentially expressed biomarkers. This parameter aims to measure the “coverage” that a certain biomarker has over the different diseases analyzed, i.e., the number of diseases it is able to distinguish.

Moreover, this research is twofold objective. The first one is the extraction of possible Differentially Expressed Genes (DEGs) that allow to discern among the different forms of leukemia and people who does not suffers from the disease. The second one is to perform a classification stage with the DEGs extracted for the assessment of those genes. Therefore, this study is about a multiclass classification problem in which a set of genes will be selected as long as they are useful to discern among the five classes: four different types of leukemia and healthy subjects. This is a very novel research because most of the studies are dichotomous, addressing only two classes for their researches. To this end, the public repository NCBI/GEO has been used [18, 19]. All the samples will be prepared for the classification step, so that the dataset that contains all the samples and only the DEGs will be used to design and assess the classifiers.

There are not many researches that combine different platforms or even different technologies with the aim of achieving a possible gene signature [20–22]. However, this work implements a pipeline with the objective of carrying out the technology integration for the extraction of the DEGs. The pipeline is an evolution of our previous research that integrates different Microarrays platform with data from skin cancer in order to be able to discern among the proposed skin cancer states [23]. Furthermore, this research also builds a set of smart leukemia classifiers to perform a differentiation among the different types of leukemia addressed when unlabeled samples are presented. To this end, the minimum-Redundancy Maximum-Relevance (mRMR) feature selection algorithm was applied in order to select the most relevant genes to improve and perform the classification [24]. Also, four different classification algorithms have been implemented and their results compared. The classifiers are the following: Support Vector Machines (SVM), Random Forest (RF), k-Nearest Neighbor (k-NN) and Naive Bayes (NB) [25–29].

This paper has been structured as follows. This section has shown the introduction and state of the art of this work. Next section explains the methodology followed in this study. It begins by describing the available data series that have been used for this research. Later, the pipeline for processing and classifying the data is shown. Next section exposes the results of the integration and the selection process for obtaining the DEGs. Furthermore, the results of the assessment of the classifiers using those DEGs will be exposed and some plots representing the expression levels of the most important DEGs will be shown. The discussion section underlines the validity of the proposed approach and its utility in the classification of unknown samples that belong to the studied types of leukemia, by using the developed machine learning tool. A biological interpretation of the DEGs selected will be exposed too. Finally, the conclusions section summarizes the most important contributions of this study for leukemia diagnosis and genetic profiling.

## Material and methods

### Samples

For this study, both Microarray and RNA-seq samples have been collected in order to accomplish the integration of a wide range of heterogeneous data. As already mentioned, all series has been downloaded from the NCBI/GEO public database. A comprehensive search has been carried out with the purpose of gathering a notable number of samples belonging to the leukemia states addressed in this work. Furthermore, as all the samples must belong to the same tissue, only samples from cells of bone marrow have been used for both healthy and leukemia samples. With regard to Microarray samples, the two main platforms (Affymetrix and Illumina) have been taken into account. For RNA-seq, samples have been solely collected from the most important sequencing platform known as Illumina HiSeq [30]. Finally, a total amount of 11 series from Microarray and 2 series from RNA-seq were selected for the research. These series are publicly available at https://www.ncbi.nlm.nih.gov/geo/query/acc.cgi?acc=S.NAME where S.NAME is the name of each series at NCBI GEO shown at Table 1, which also includes information about the collected series.

**Table 1:** Relevant information about the series studied in this research. *Total Samples* column represents the total amount of samples that each series contains. *Accepted Samples* column denote the number of samples that belong to the different leukemia or healthy states and that will be analyzed in this study. The *Outliers* column quantifies the low quality samples that were removed from the *Accepted Samples*. Finally, the *Procedence* column reveals the genetic diversity in the origin of the series for thus study.

Series	Platform	Technology	Total Samples	Accepted Samples	Outliers	Procedence
GSE6691	Affymetrix Human Genome U133A Array	Microarray	56	11	0	Salamanca (Spain)
GSE51082	Affymetrix Human Genome U133A Array	Microarray	139	55	1	Oregon (USA)
GSE12417	Affymetrix Human Genome U133A Array	Microarray	163	152	11	Munich (Germany)
GSE21029	Affymetrix Human Genome U133 Plus 2.0 Array	Microarray	62	19	1	Bethesda (USA)
GSE49067	Affymetrix Human Genome U133 Plus 2.0 Array	Microarray	12	12	0	Boston (USA)
GSE36474	Affymetrix Human Genome U133 Plus 2.0 Array	Microarray	7	3	0	Brussels (Belgium)
GSE33075	Affymetrix Human Genome U133 Plus 2.0 Array	Microarray	27	24	3	Salamanca (Spain)
GSE34860	Affymetrix Human Genome U133A Array	Microarray	78	78	0	Milan (Italy)
GSE61853	Illumina HumanHT-12 V4.0 expression beadchip	Microarray	14	7	0	Daejeon (South Korea)
GSE11504	Affymetrix Human Genome U133 Plus 2.0 Array	Microarray	25	7	0	Oslo (Norway)
GSE13576	Affymetrix Human Genome U133 Plus 2.0 Array	Microarray	209	197	0	Padova (Italy)
GSE98310	Illumina HiSeq 2000	RNA-seq	22	22	0	Montreal (Canada)
GSE63646	Illumina HiSeq 2500	RNA-seq	71	71	0	Columbus (USA)
**TOTAL**	-	-	**885**	**658**	**16**	-

Additionally, Table 2 shows the number of samples of each class for each gene quantification technology. It is worth noting that only Microarray technology presented samples for each of the studied classes. Indeed, there are not enough public samples of these states for RNA-seq. This is an important motivation to keep using Microarray samples and take advantage of them. In this sense, the integration that our pipeline performs is a significant step forward.

**Table 2:** Number of categorized samples collected for each of the applied gene quantification technologies. HBM stands for *Healthy Bone Marrow* and the rest represent the four types of leukemia. A lack of RNA-seq samples is clearly showed except for the AML state.

Type/State	HBM	AML	ALL	CML	CLL
**Microarray**	26	259	197	53	29
**RNA-seq**	0	93	0	0	0
**Total**	26	352	197	53	29

### Tools

The two main tools used for the development of this study have been R and Matlab [31]. On one hand, R was used for the multiclass DEGs extraction. A group of packages from Bioconductor were also used in R and they will be explained described in detail in the pipeline explanation below [32]. On the other hand, Matlab was used to apply the mRMR algorithm to create and assess the classifiers.

### Proposed pipeline

**Fig 1:**
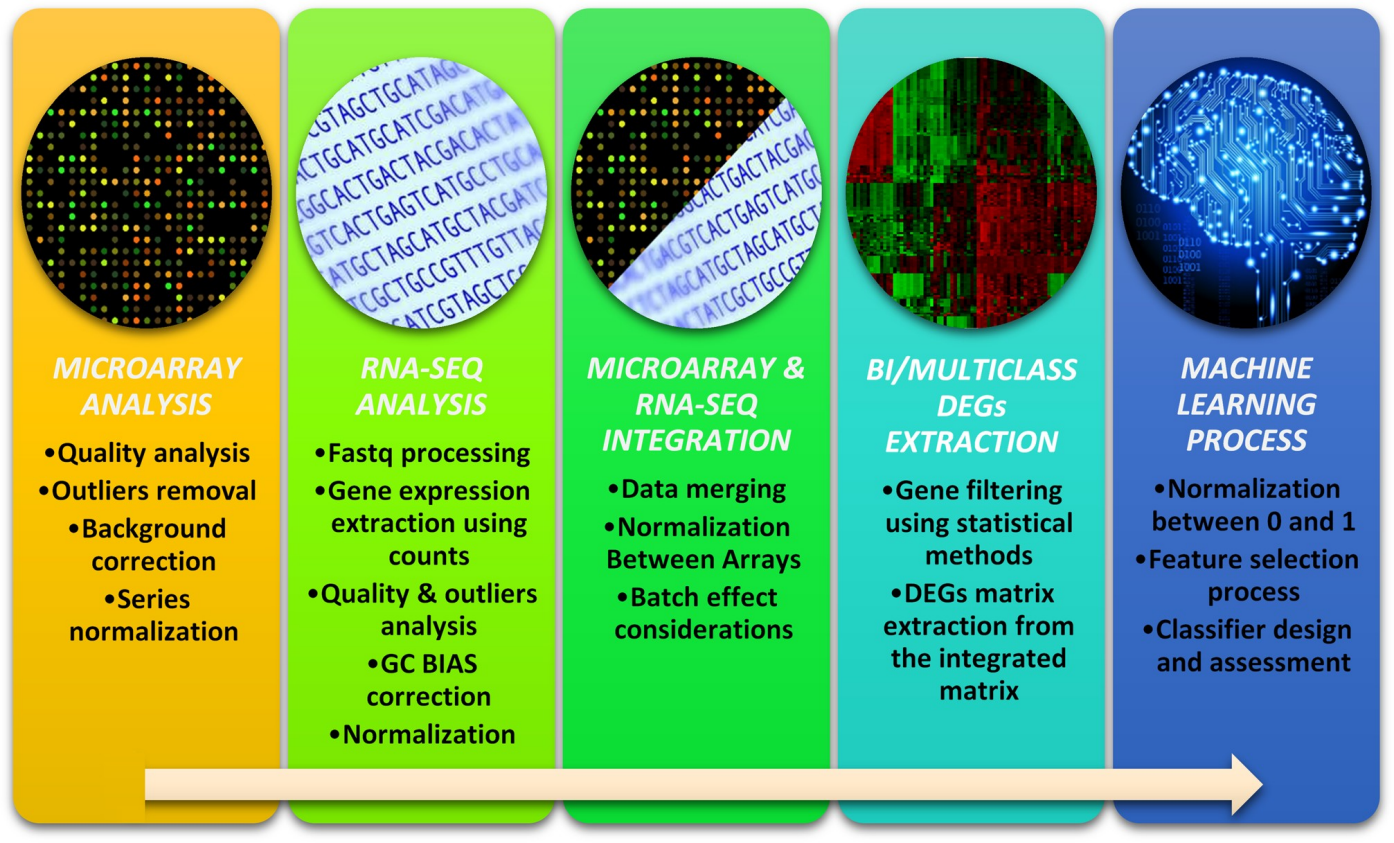
Proposed pipeline for the correct integration and classification of heterogeneous (Microarray and RNA-seq) biological data.

In this subsection, the pipeline followed in this study is explained in detail. The pipeline can be split into four steps or parts, as it is shown at Fig 1.

#### Microarray analysis

The first step is the analysis of each Microarray that will be used in the study. This step is valid for any Microarray technology, and in this work, it will be applied to the Microarrays from Affymetrix and Illumina.

Firstly, it is very important to carry out a quality analysis of the available samples in the selected series in order to detect and consequently remove any possible outlier. This outliers detection and removal was performed through *arrayQualityMetrics* R package, which computes the Kolmogorov-Smirnov statistic *K*_*a*_ between the distribution of each array and the distribution of the pooled data [33]. Secondly, once there are no remaining outliers, a normalization step is required. In order to perform this normalization, the *rma* function from *affy* bioconductor package was used for Affymetrix Microarrays [34]. In the case of Illumina Microarrays, *lumiExpresso* function from *lumi* bioconductor package was used [35]. Both functions perform not only the normalization but also the background correction for each microarray series.

#### RNA-seq analysis

The second step in the pipeline corresponds to the RNA-seq analysis. This step follows the same ideas of the Microarray analysis except for a few differences, being the most relevant variation the data extraction process due to the necessity of a whole pipeline only to transform the raw data of RNA-seq into gene expression values.

This RNA-seq processing pipeline was proposed by Anders et al. [36]. Starting from the SRA original files, several tools like *sra-toolkit*, *tophat2*, *bowtie2*, *samtools* and *htseq* have been used in order to obtain the gene count files for each sample [37–41]. Once the read count files were obtained, the gene expression values were calculated by using the *NOISeq* R packages [42].

For the quality analysis and outliers detection, the same pipeline followed in Microarray can be used if the gene expression values are available. Furthermore, for the normalization and GC BIAS correction, the function cqn from *cqn* package was used [43]. Moreover, due to the nonexistence of replicates in this study, no process of normalization between replicates was applied.

#### Microarray and RNA-seq integration

The Microarray and RNA-seq integrative pipeline merges data both from Microarray and RNA-seq technologies. There are two important steps for an appropriate integration of heterogeneous data: data merging and normalization.

The first step, data merging, requires the correct individual pre-processing of all available series in the study. Afterwards, heterogeneous integration was carried out using the *merge* function from the base R package.

The second step in the integration is the normalization of the integrated dataset. To perform this normalization, the *normalizeBetweenArrays* function was used [44]. This function is based on quantile normalization and allows to remove any possible deviation or variation among different arrays or datasets. Concretely, the method selected for the normalization in this function is the “Aquantile” that ensures that the A-values (average intensities) have the same empirical distribution across arrays, leaving the M-values (log-ratios) unchanged” [44].

These tasks are essential in order to achieve a correct normalization of the biological data and its subsequent processing [45, 46]. The integration is indeed a critical process in the study as if the merging or normalization are not properly done then the extracted DEGs would be erroneous. This would introduce confusing values in the research, that would lead irretrievably to a misleading selection of DEGs and subsequent erroneous outcome of the machine learning process.

With regard to the batch effect, it was addressed by using the considerations explained by Goh et al. [47] having into account the important unbalancement that exist among the batch groups of the different series of this study.

#### DEGs extraction

Once the integration has been properly done, the next step is the DEGs extraction. There are some statistical toolboxes/routines that allow to estimate if a gene has enough statistical significance for being considered a DEG or not.

As mentioned before, a multiclass problem is addressed in this study, which means that DEGs have to be valid for discerning among more than two classes. Specifically, five classes are identified in this study, being necessary to find a group of biomarkers with the capability of discerning among these classes in order to achieve multiclass classification. However, it is important to notice that limma, when dealing with a multiclass problem, takes into account only one value of the LFC between two classes, to identify if a gene is relevant or not, omitting the needed consideration of the rest of classes and class comparisons.

The total number of binary problems (taking one against one class comparisons) that take place in a problem with N classes can be defined as shown in Eq 1. With the purpose of properly identifying the differences in gene expression among all classes involved in a multiclass problem, and thus identifying the best DEGs for the same purpose, we will define the COVERAGE (COV) of a gene, as the number of class pair comparisons that a gene covers when a LFC restriction is imposed (furthermore, the possible DEGs also have to reach a p-value lower or equal to 0.001). Then, Eq 1 also represents the maximum number of binary problems/comparisons about which a certain biomarker provides information, so it will be called from now on *COV*_*max*_. In our present problem including 5 classes, *COV*_*max*_ takes the value of 10 (ten pairs of class combinations). The real potential of this parameter lies in the ability to discover high coverage DEGs, thus allowing us to discern among the maximum possible number of classes.

$$\begin{array}{c} COV_{max}=\frac{N^{2}-N}{2} \end{array}$$ (1)

Using *COV* as criteria to identify gene signatures implies choosing a coverage threshold, so that a certain biomarker will be selected only if it covers or differentiates at least a certain number of binary class comparisons. A large coverage threshold could be too restrictive, and a small coverage threshold could lead to the selection of too many biomarkers. The determination of an appropriate *COV* threshold is therefore critical in this problem. A medium size *COV* representing a trade-off between large and low differentiability in number of class pairs -coverage- (a fraction of *COV*_*max*_, such as *COV*_*max*_/2, *COV*_*max*_/3, depending on the number of classes considered) may seem to be a reasonable threshold. The ANOVA test presented later will study the performance of different *COV* threshold values.

Finally, all the genes that pass the restrictions imposed by the p-value, LFC and COV restrictions will be selected as DEGs for discerning among the five classes of this study.

#### Machine learning process

DEGs extraction is a very sensitive process because an error in the parameters or in the design of the followed pipeline could lead to a mistake in the right identification of the DEGs. In order to assess the DEGs selection, a machine learning process is performed. This process is explained below.

Firstly, the DEGs dataset is normalized with median 0 and standard deviation 1. This step homogenizes into the same type of distribution the gene expression values and can suppress the effect of possible remaining outliers due to the bounded range.

Although the use of limma for the extraction of DEGs already identifies candidate genes for the classification of the different diseases, the application of a specific feature selection process before the classification step would reduce the size of the DEGs set, thus simplifying the classification and even improving the final accuracy. Precisely, the mRMR algorithm is used in order to obtain a ranking with the most appropriate combination of our DEGs, according to the operation of the algorithm. To create this ranking, mRMR takes into account the relevance and redundancy information among the genes. Using both criteria, mRMR sorts the genes so that they bring largest relevance with respect to the class and, at the same time, they have lowest redundancy among themselves. Therefore, this algorithm will rank in first position the gene that contains the largest amount of information, but the following genes will provide also minimum redundancy (apart from maximum relevance as regard to the class) with respect to the already selected genes.

When both, the normalization and feature selection process are done, four different supervised learning algorithms that will be exposed below are applied to an increasing number of selected DEGs, according to the mRMR algorithm. A cross-validation step using k-fold is performed in order to assess the results of the classifiers on the dataset. K-fold cross-validation algorithm iteratively leaves out 1/k data from the training dataset, which are used to assess the classifier when the training is done. Finally, both accuracy and f1-score are calculated using the outcomes from the k assessment processes. This last measure takes into account the grade of classification of each class, not only the total amount of samples correctly classified. Eq 2 represents f1-score, that is used when a multiclass problem is tackled due to the relevance of this measure in this type of problems. It is calculated by using both the precision or accuracy and the recall or sensitivity.

$$\begin{array}{c} f1\_score=2*\frac{precision*recall}{precision+recall} \end{array}$$ (2)

The whole machine learning process has been explained so far, but without going into detail of each of the four implemented classifiers: Support Vector Machine (SVM), k-Nearest Neighbor (k-NN), Naive Bayes (NB) and Random Forest (RF). These are shortly described next.

- SVM: The idea of SVM is the selection of an hyperplane that is equidistant from each class, thus achieving a maximum-margin for the separation of the classes. Moreover, when the hyperplane is defined, only the samples that fall into the frontier are taken into account as training support vector samples. Furthermore, the algorithm tolerates classification errors, which are controlled by the *γ* hyperparameter, controlling the generalization capability of the model [25, 26]. Finally, for a biclass classification a new sample will be classified depending on the side of the hyperplane to which this sample belongs. However, for a multiclass classification the method changes because SVM builds (N-1)*N/2 classifiers (where N is the number of classes) and it establishes a voting system among them in order to decide which is the most voted class for the new samples.
- k-NN: This algorithm assigns to a new unseen sample, the predominant class corresponding to the k nearest neighbors (most similar samples) using the known labeled data. It is a well-known fast and easy-to-use technique which however may provide a comparable performance to other well-known more complex techniques [28, 48].
- NB: A naive Bayes classifier assumes that each characteristic is independent to the rest. Therefore, each of the DEGs contributes in an independent way to the probability of being part of a particular class. A clear advantage of this type of classifiers is that these need a small number of training samples for estimating the necessary parameters for the classification (mean and variance). It is a very fast and efficient classifier for supervised learning problems such as the problem addressed in our study [49].
- RF: Random Forest algorithm grows many single classification vector (trees) with the purpose of building a forest of classification trees. For the classification, the algorithm assigns a vector as input to be classified for each tree of the forest. Once that each individual tree performs the classification, a voting system among the trees decides the class having the largest number of votes over all the trees [27, 50].

### ANOVA test

The ANOVA test plays a very important role in this study. It will analyze and compare the performance of the four classifiers, as well as of different combinations of the hyperparameters LFC and COV for optimal relevant biomarker identification. This step is very useful in order to determine if the classifiers have significant differences among them. Moreover, the test will provide valuable information about which combination of LFC and COV is better for our study and it will bring some light into the optimization of these parameters for further studies.

## Results

This section will be split into three subsections in order to expose the results in the most clearly and organized way. In the first subsection, the impact of both the LFC and the COV on the final classification results is evaluated performing an ANOVA test. In the second subsection, the first four steps of our pipeline are applied for the extraction of the DEGs. Finally, the last subsection and also the last step in our pipeline is the DEGs ranking process, and the assessment of those DEGs using a machine learning process.

### Statistical analysis: ANOVA test

The ANOVA test is very important at this point due to two main reasons. Firstly, there are several possible values that both the LFC and the COV could take. Secondly, in this research, four different classifiers are assessed. In view of these considerations, the test can decide if there are statistically significant differences among the classifiers and how the value of both the LFC and the COV could affect final results. The chosen classifier, LFC and COV are the parameters that a priori could cause more impact in the study. Nevertheless, the final number of selected genes after a feature ranking process -mRMR- could also affect final results, so it will also be considered.

Therefore, the possibles values that the four factors can take in the test are the following:

- Classifier: this variable represents the classifier used for the simulation. This classifier can be SVM, k-NN, NB or RF.
- LFC: this variable represents the Log-Fold Change used in order to extract the relevant genes, taking the values 1, 1.5, 2, 2.5 or 3.
- COV: this variable represents in a multiclass problem, the number of combination of classes in which a gene is truly relevant, taking the values 2, 3, 4 or 5.
- NR. GENES: this variable represents the number of genes finally selected with mRMR, which were used as input for the classifier, taking values 10, 20, 30 or 40.

From all the possible combinations of the former four factors, a wide range of simulations have to be addressed and evaluated in order to achieve an statistical interpretation of the results. Consequently, the test evaluates how both accuracy and f1-score are affected by the four chosen variables for the test (Classifier, LFC, COV, number of genes). Table 3 shows the relevance of these variables with respect to the accuracy. In this table is clearly seen how all the studied variables are relevant with regard to the accuracy. This means that it is important to take all of them into account for the research.

**Table 3:** Variance analysis for the accuracy—Sum of Squares type III.

Source	Sum of Squares	Gl	Medium Square	F-value	P-value
**Mainly effects**					
**A: Classifier**	0.0802257	3	0.0267419	67.86	0.0000
**B: LFC**	0.00438299	4	0.00109575	2.78	0.0256
**C: COV**	0.00660603	3	0.00220201	5.59	0.0008
**D: N° GENES**	0.0724379	3	0.024146	61.27	0.0000
**Residuals**	0.593102	1505	0.000394088		
**Corrected Total**	0.757276	1518			

In the same way that the previous table shows how selected variables affect accuracy, Table 4 analyzes the impact on the f1-score. As it happened for accuracy, selected variables are also relevant for the f1-score.

**Table 4:** Variance analysis for the f1-score—Sum of Squares type III.

Source	Sum of Squares	Gl	Medium Square	F-value	P-value
**Mainly effects**					
**A: Classifier**	0.0177814	3	0.00592712	38.60	0.0000
**B: LFC**	0.010959	4	0.00273975	17.84	0.0000
**C: COV**	0.0014741	3	0.00491365	32.00	0.0000
**D: N° GENES**	0.0398155	3	0.0132718	86.43	0.0000
**Residuals**	0.230789	1503	0.000153552		
**Corrected Total**	0.311559	1516			

**Fig 2:**
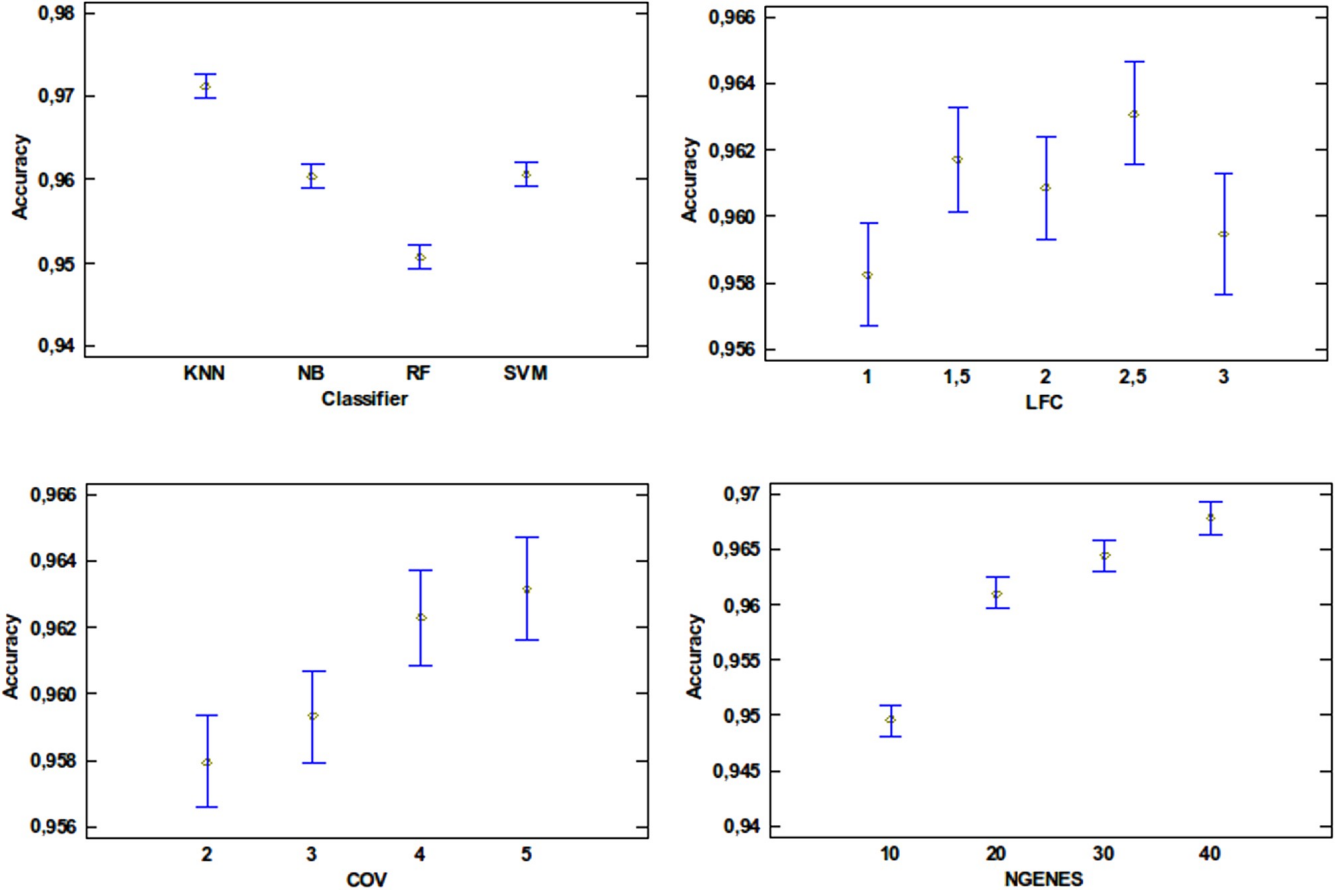
ANOVA results showing the impact on accuracy for each of the evaluated variables.

Previous tables showed how the four studied variables affected both accuracy and f1-score. At this point, the impact of each value that the different variables could take will be described through a group of plots. In this sense, Fig 2 shows a graph for each of the studied variables (Classifier, LFC, COV, NR. GENES). The classifier variable chart clearly shows that the classifier attaining the highest accuracy is k-NN. Furthermore, both SVM and NB obtain the same results leaving RF as the worst classifier for this study.

In the case of LFC, the plot shows that an increase of LFC does not lead to an accuracy improvement. However, it is really important to note the behavior of COV. As it can be seen, an increase in COV leads to an accuracy improvement, meaning that the new proposed measure is an important criterion in the selection of multiclass biomarkers. Among the values compared, ranging from 2 to 5, an improvement in the final recognition was shown due to the fact that as the value of COV increases more classes are covered by the selected genes.

Finally, it is straightforward to expect that larger gene signatures may attain higher accuracy in the identification of the different pathologies studied than shorter gene signatures. This is shown by the NR. GENES variable, whose values range from 10 to 40.

**Fig 3:**
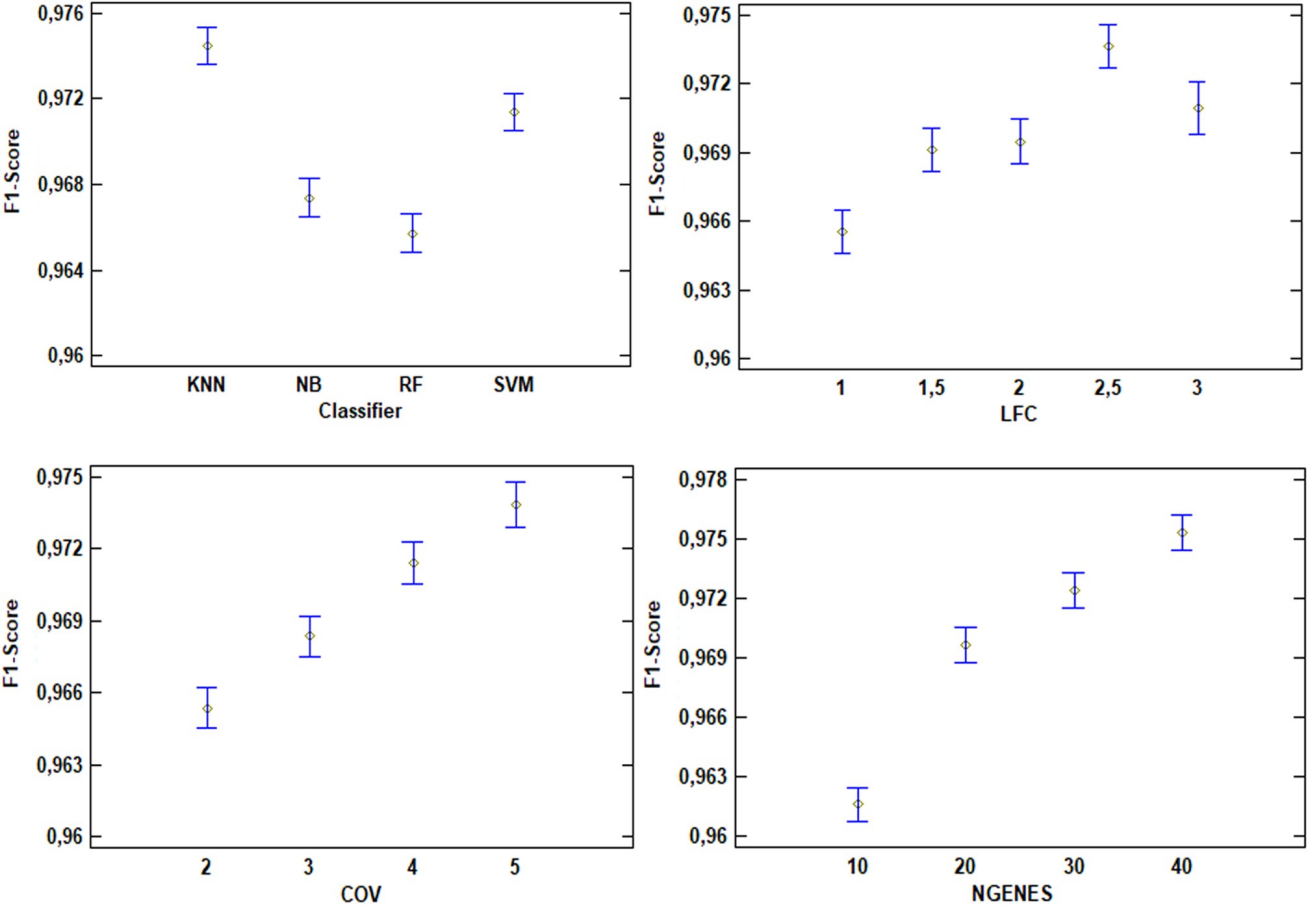
ANOVA results showing the impact on the f1-score for each of the evaluated variables.

In the same way that the previous figure shows how the variables have a direct impact in the accuracy, Fig 3 represents the impact regarding the f1-score. The results for the LFC, COV and NR. GENES have the same behavior in the f1-score than in the accuracy but for the classifier variable. In this case, SVM achieves better results than the NB. However, k-NN is still the best classifier and RF the worst for the study.

At sight of the ANOVA test results, only one of the possible combinations of LFC and COV will be taken into account to extract the biomarkers of this study. Specifically, the LFC equal or greater than 2.5 and the COV equal or greater than 5. These values are both the best combination in terms of achieving the best possible results, as can be seen in the ANOVA plots exposed before. Then, for these requirement settings, gene signatures with different NR. GENES will be finally studied, and the detailed results of the four classifiers will be shown.

### Differential expressed genes extraction

Once the ANOVA test was performed and specific values for both the LFC and the COV were selected, we will proceed to extract the DEGs using these values.

**Fig 4:**
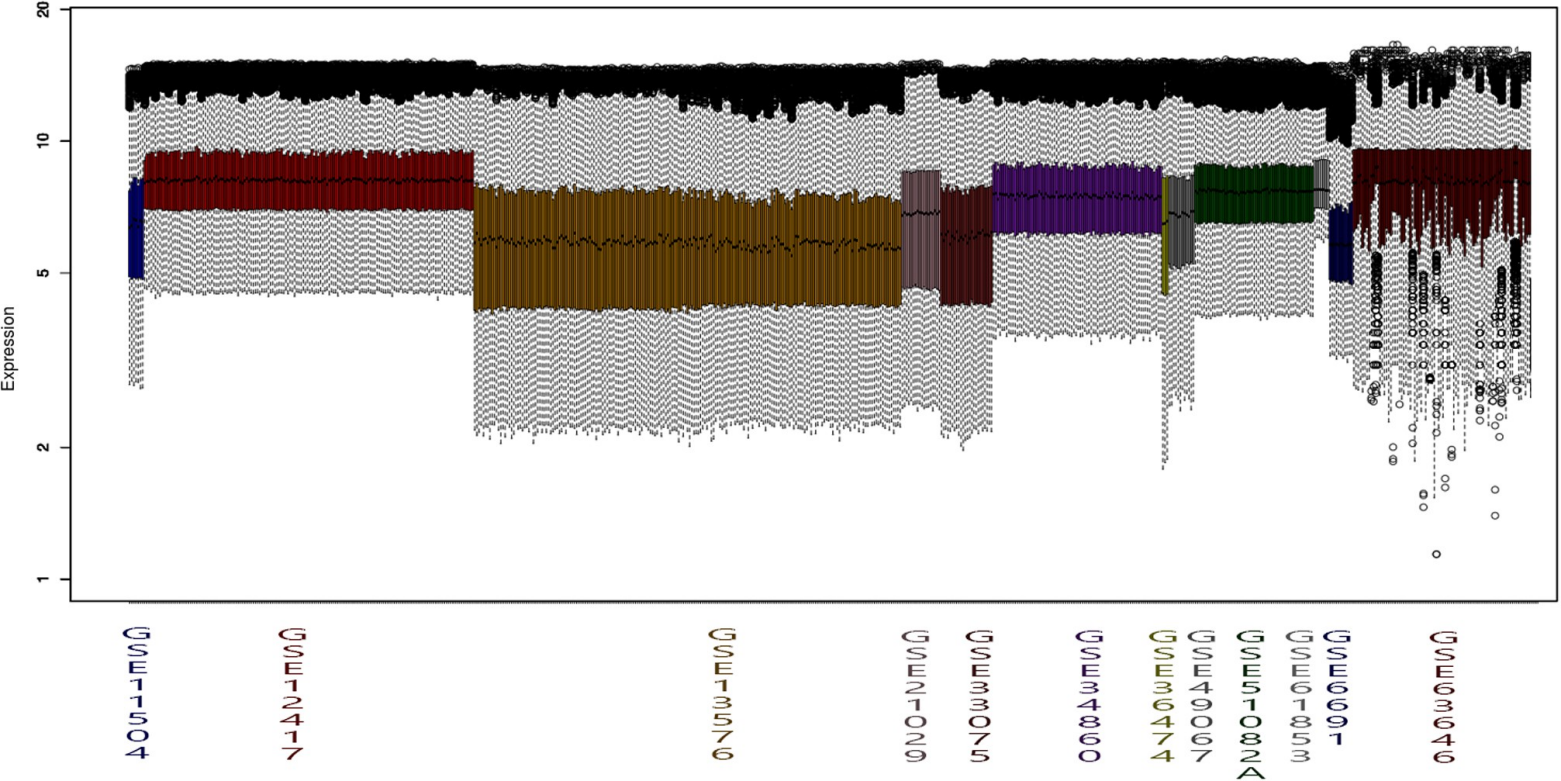
Expressions values comparison among leukemia series before the joint normalization and integration steps.

However, before presenting the DEGs extracted, the results of the series integration for this study will be shown. In order to perform an integrated analysis of these series, an individual analysis and correction of each series was done. Furthermore, it is necessary to correct the existing differences among the series due to the variety of technologies and platforms present in this study. In this sense, Fig 4 shows the normalization and bit depth correction across the series once have been normalized separately.

**Fig 5:**
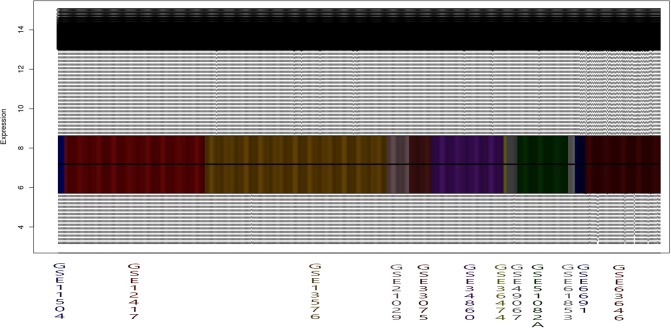
Expressions values comparison among leukemia series after the joint normalization and integration steps.

In order to achieve the best coercing among the series after the integration, a joint normalization with quantile normalization is required, with the purpose of obtaining the same dynamical range among the series (see Fig 5).

Once normalization using the *normalizationBetweenArrays* function has been accomplished, the dataset is completely ready to perform gene expression analysis. Thus, limma will be used in order to achieve this analysis. Nevertheless, it is important to note that this study is a multiclass gene expression problem. Therefore, it is necessary to use limma with this consideration, avoiding the classical biclass pipeline which limma implements by default.

Once multiclass limma was applied, a list of DEGs were reported. Those genes passed the three imposed restrictions: LFC equal or greater than 2.5, COV equal or greater than 5 and p-value equal or less than 0.001. A total of 42 genes that satisfy these restrictions were returned. Table 5 collects the 42 genes and shows statistical values about those DEGs. These statistical values are the T-statistic, the P-value, the LFC and the COV for each gene.

**Table 5:** Table with the expressed genes that represents several statistical values of these genes.

Expressed Genes	| *μ T* − *statistic*|	| *μ P* − *value*|	| *μ LFC*|	COV
HIPK1	16.0875204	2.4713e-23	3.288872	6
SLC4A1	13.5735052	4.7010e-12	3.421740	6
FGD2	22.0274586	8.1678e-21	4.201152	6
DOCK2	24.4474942	1.9231e-24	3.678663	6
PADI2	22.1425831	1.3321e-30	3.050733	6
LAPTM4B	10.7437126	1.1139e-12	2.935885	6
C11orf58	12.5259082	6.9347e-10	4.240850	6
LITAF	12.7181709	2.1436e-10	3.927194	6
SF1	10.9802843	4.1383e-06	4.076528	6
RPS24	9.6354228	5.2593e-07	3.943997	6
CLEC2B	8.5098540	1.2060e-11	3.407249	6
EEF1A1	10.8653720	9.2231e-08	4.247919	7
LGALS3	11.2588096	1.2091e-10	3.999583	6
PLCG2	28.0170676	1.6955e-56	3.616019	6
BANK1	17.9033482	1.2248e-26	4.082236	6
H2AFY	13.7470887	2.1613e-09	5.151885	6
TSPAN3	18.6133699	1.8746e-18	3.606852	6
MKNK1	14.3396635	8.2579e-12	3.432306	6
PABPC1	9.7297018	2.8194e-06	4.177165	6
TAB2	15.0096557	3.7740e-18	3.071626	6
ROCK1	23.5206238	2.6058e-34	3.951989	6
RPS15	19.3210248	1.1963e-10	5.208190	7
GSN	8.5034573	3.6481e-10	2.994001	6
CMTM6	13.4600508	2.1206e-08	4.303823	6
FUS	22.7727271	1.5430e-33	3.524736	6
SEPT7	20.3301923	6.7129e-24	3.804574	6
ZNF160	14.9926210	5.0560e-20	3.280500	6
ANXA2	10.3112336	1.0576e-07	4.099294	6
EAF2	11.5559113	1.4391e-09	3.466531	6
TCF4	15.7648662	2.0821e-22	3.698245	6
CD22	34.1927437	1.1397e-64	3.522825	6
POU2AF1	31.2157033	8.9199e-59	5.590414	6
CFD	16.3828557	2.5946e-18	3.855234	6
BLK	34.6994030	2.5379e-69	5.076078	6
CD19	36.4443927	6.559e-108	4.806683	6
BLNK	25.7630631	1.7997e-37	5.563086	6
ACTN1	14.7867854	2.1587e-20	4.260679	6
CTGF	11.8350681	7.5825e-13	4.142035	6
TCL1A	24.0024506	1.1794e-34	4.774254	6
PPP1R16B	11.0936657	4.0266e-11	2.949562	6
AZU1	11.1121946	4.3126e-11	4.161520	6
ATP8B4	17.1102708	2.0803e-28	3.665865	6

### Genes assessment using a machine learning process

Once the DEGs extraction pipeline was done by using the integrated dataset, the feature selection algorithm mRMR was applied, obtaining a ranking of genes in which the most relevant genes would be placed on the first positions within of this ranking. Furthermore, thanks to its operation taking into account the mutual information among the selected genes, this algorithm can also minimize the redundancy among them.

**Fig 6:**
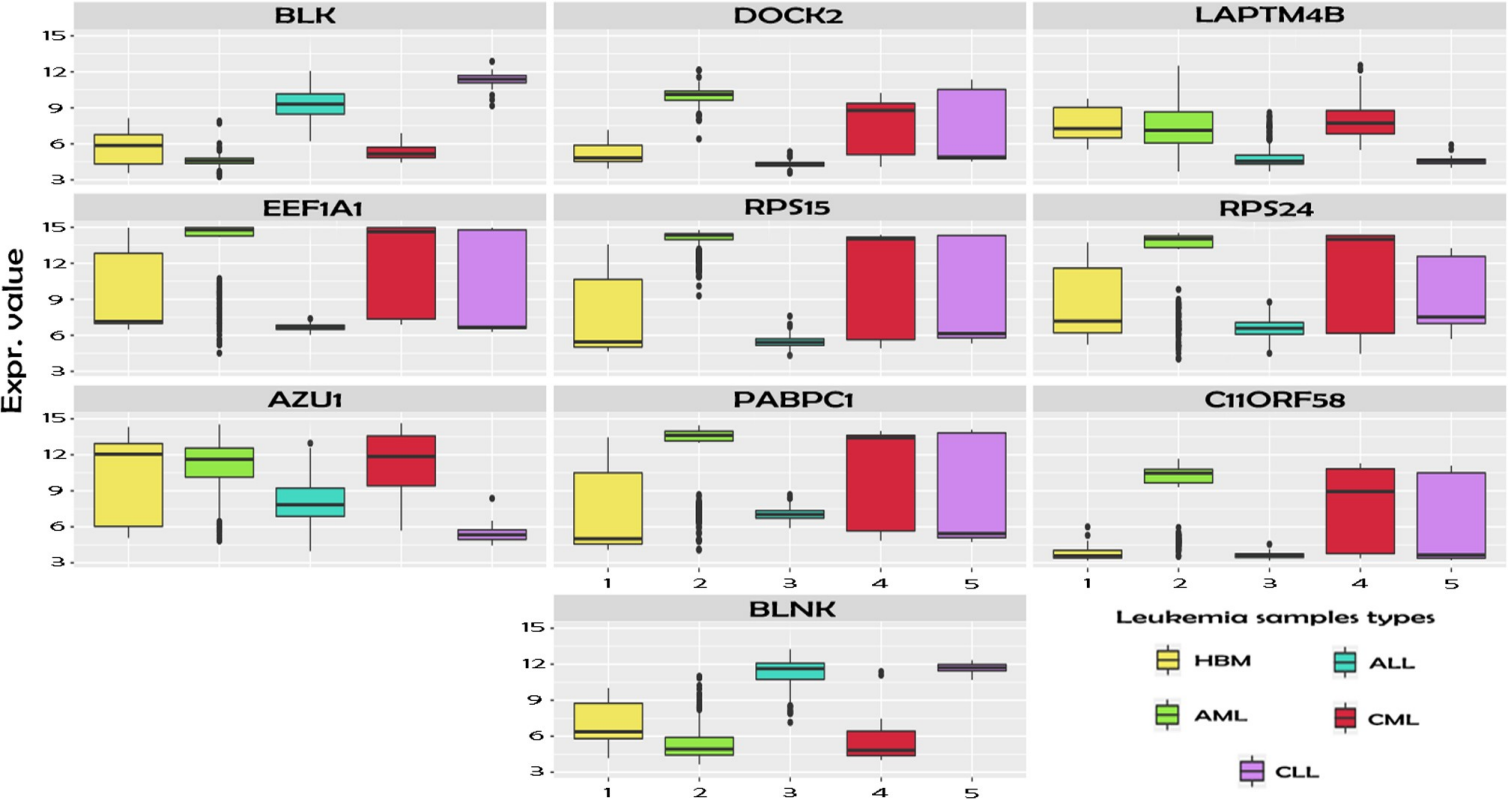
10 first selected differentially expressed genes by mRMR algorithm (order from left to right and from top to bottom: BLK, DOCK2, LAPTM4B, EEF1A1, RPS15, RPS24, AZU1, PABPC1, C11ORF58 y BLNK), with the expression levels for each type of leukemia studied.

Fig 6 shows in an ordered way the 10 first genes returned by mRMR ranking, revealing for each of them, the expression levels of each class. Such genes will be more deeply commented both at bioinfomatic and at biological level in the Discussion section. Reminding the COV parameter introduced in this study, it can be observed how the DEGs, concretely the 10 first of the mRMR ranking, present different expression levels not only for one class with regard to the others but also among several classes. For example, the first gene of the Figure (BLK) shows different expression levels for four of the types of leukemia, hence allowing us to discern among these classes. Indeed, due to the behavior of the mRMR algorithm, this gene is the one with the highest level of Mutual Information with respect to the classifier variable.

Subsequently, the performance of the obtained ranking was evaluated. For that, four different classifiers were implemented and compared. Furthermore, this comparison has been performed for different number of genes (10, 20, 30 and 40) and for both the ACC and the f1-score. As for the simulations of the ANOVA test, a cross-validation process (5-fold) was applied with the objective of providing an estimation of the performance on unseen samples, avoiding overfitting. This restrictive cross-validation process guaranteed a significant representation of the lowest frequent classes (specially HBM and CLL, see Table 2) in all data folds.

**Fig 7:**
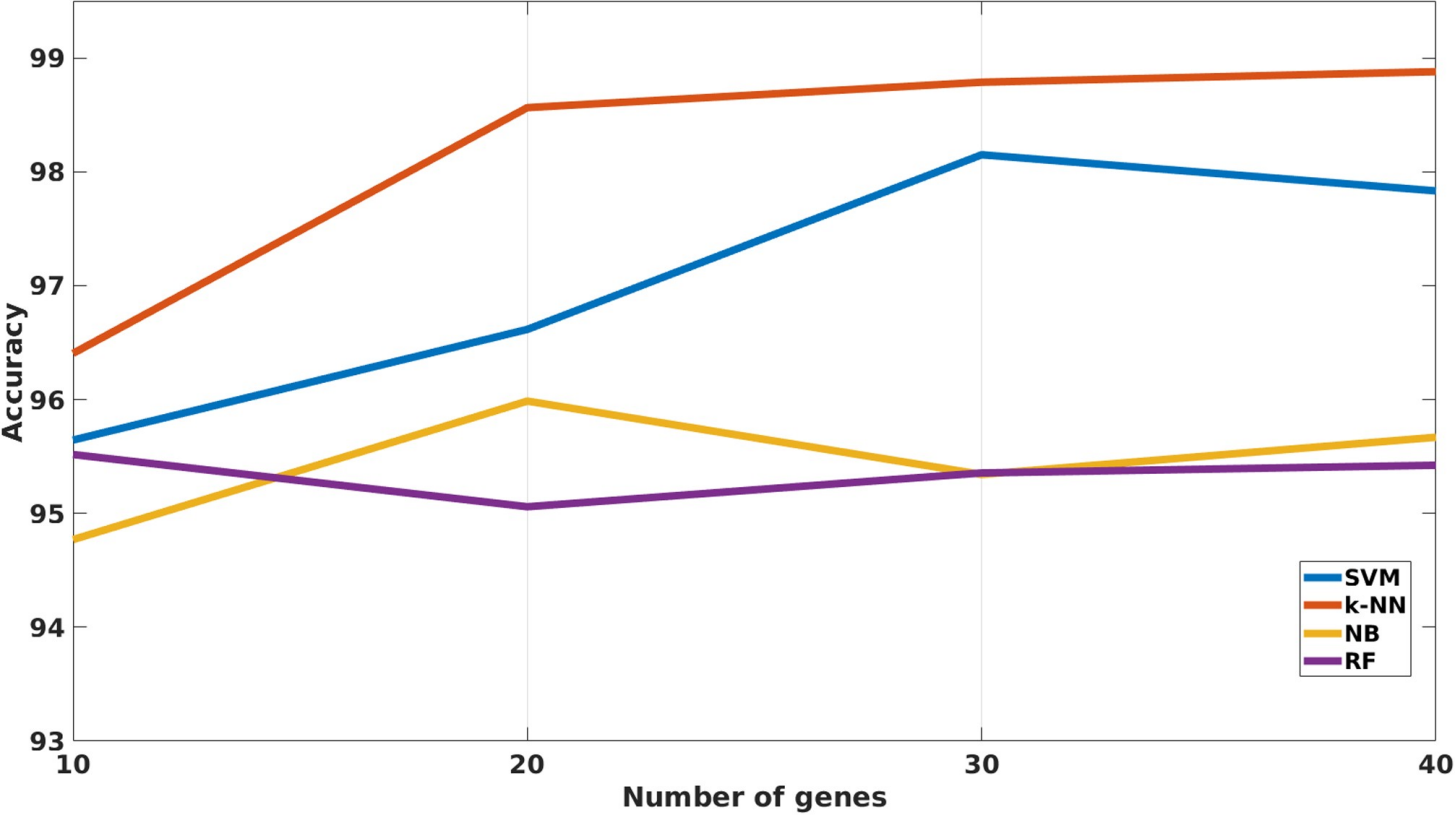
Plot that represents the accuracy achieved by each of the four classifiers used in the study.

**Fig 8:**
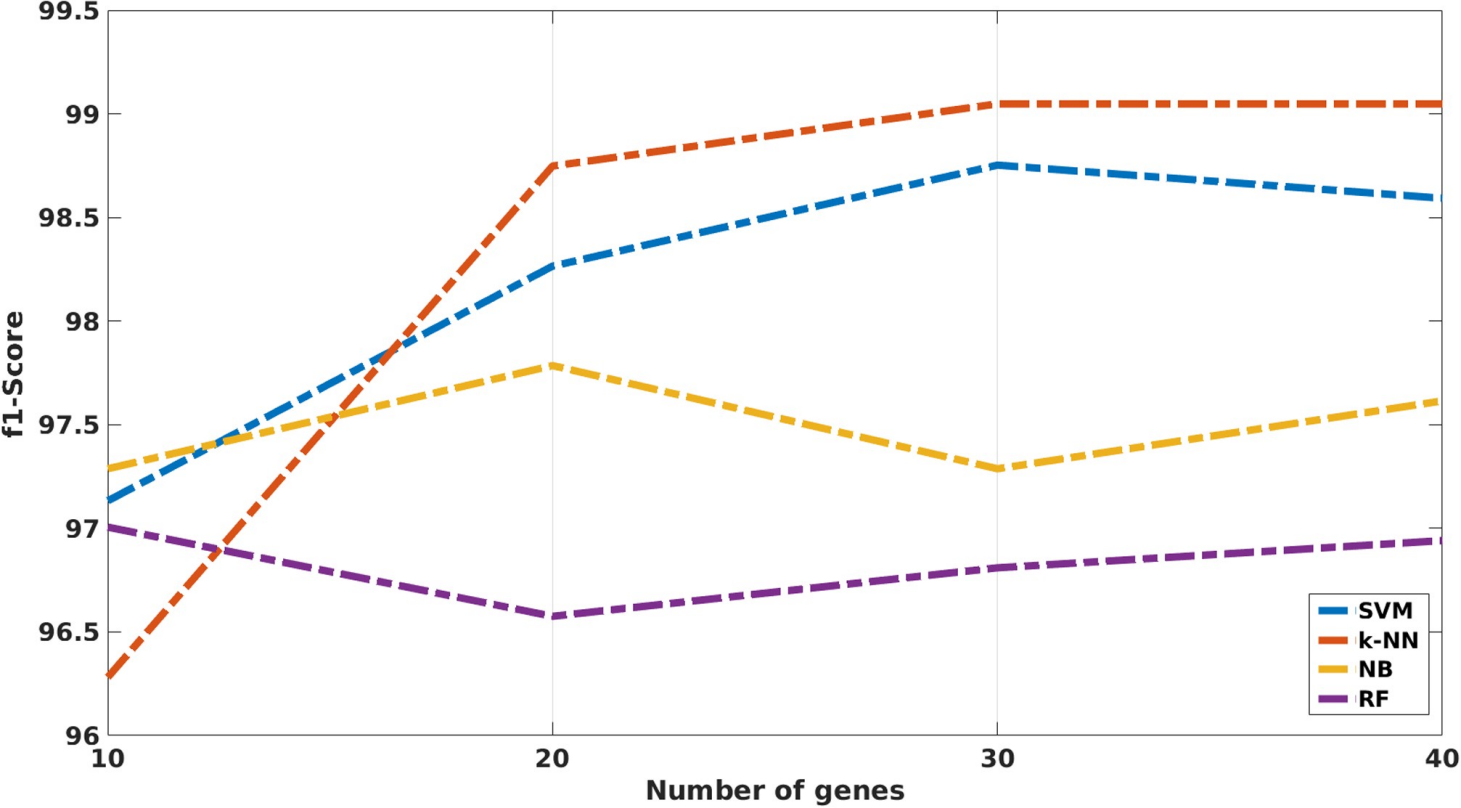
Plot that represents the f1-score achieved by each of the four classifiers used in the study.

The result of these comparisons can be seen at Table 6. This Table shows how k-NN reaches better results with respect to the rest of the classifiers in practically all the comparisons, reaching 96.40% of ACC using only the 10 first DEGs chosen by mRMR algorithm, from the total of 42 DEGs. However, the f1-score reached by k-NN in this case is lower than the one reached by the rest of the classifiers for this number of genes. For 20 DEGs, k-NN reaches 98.56% of ACC and 98.75 of f1-score, being ahead of the rest of the classifiers. This behavior is repeated for both 30 and 40 DEGs as can be seen at Fig 7 and at Fig 8. These figures show the evolution of the ACC and the f1-score, respectively, for the four implemented classifiers. Regarding the rest of classifiers, SVM reaches comparable although slightly worse results than k-NN for 30 and 40 genes. Finally, RF and NB present clearly worse results regardless the number of selected genes.

**Table 6:** Results of the four classifiers for both the accuracy and f1-score when using a different number of genes.

	*10 Genes*		*20 Genes*		*30 Genes*		*40 Genes*	
Classifier	ACC	f1-score	ACC	f1-score	ACC	f1-score	ACC	f1-score
**SVM**	95,64%	97.13%	96,61%	98.27%	98,14%	98.75%	97,83%	98.59%
**k-NN**	96.40%	96.28%	98.56%	98.75%	98.78%	99.05%	98.87%	99.05%
**NB**	94.76%	97.29%	95.98%	97.79%	95.34%	97.79%	95.66%	97.61%
**RF**	95.51%	97.01%	95.05%	96.58%	95.35%	96.81%	95.42%	96.94%

## Discussion

Once the results of this study were exposed, the discussion of these will be performed with the purpose of giving an explanation both at biological and bioinformatic level.

### ANOVA test statistical interpretation

ANOVA test involves one of most important and crucial step carried out in this study due to the relevance of the parameters involved in the test. All the conclusions acquired emanated from the interpretation of both Tables 3 and 4 and also from both the Fig 2 and the Fig 3.

Firstly, the best classifiers for both the ACC and the f1-score were k-NN and SVM. This behavior is the same one observed in our previous study related to breast cancer but at bi-class level instead of at multiclass level [17]. This result also coincides with the results obtained in other related works [51–53]. Furthermore, a Naive Bayes classifier was implemented in order to assess its performance with respect to the other classifiers, which were already compared in the literature. However, the performance obtained by the NB classifiers came out to be worse than SVM and k-NN classifiers. Both, SVM and k-NN work based on distances or similarity measures (kernels), while NB is based on bayesian probabilities and RF is based on the creation of decision trees to carry out the classification. This operation of both k-NN and SVM algorithms might be giving rise to these results.

Secondly, it was observed how the LFC parameter does not have a relevant impact on the optimal DEGs selection. For the evaluated values of this parameter, there is not a clear increase of the performance when the value of LFC increases too. Nevertheless, it is important to note that LFC is not used in a isolated way as this parameter is used in conjunction with the COV parameter, which showed to be the most important parameter for the DEGs selection.

Thirdly, the statistical evaluation of the COV parameter proves that it is the most important parameter in this study. COV parameter increases the classification performance as its value increases too. However, this parameter must be used carefully in a multiclass problem. A too high value of the COV parameter can lead to an aggressive and extreme reduction on the final number of selected DEGs. On the other hand, a too small value of this parameter would let too many genes, increasing the payload of the system and reducing the possibility of finding a reduced and thus useful genetic signature. In our case, the ANOVA test shows how an intermediate COV value of 5 was the optimal among the studied values and hence, this value was used for the definitive extraction of the DEGs.

Lastly, when the number of genes increases, the final classification rate increases too for both the ACC and the f1-score measures. Nevertheless, this rise significantly decreases when the number of genes exceeds the value of 20, showing either that it is not possible to provide more information to perform the classification, or that overfitting occurs.

To sum up, it can be seen how the ANOVA test shows the importance of the considered parameters, excluding the LFC, for the optimal recognition of possible genetic signatures. Moreover, thanks to the new parameter COV great classification results were obtained due to the multiclass DEGs selection achieved by this parameter.

### Differential expressed genes selection and assessment

The ANOVA test allows defining an optimal parametrization for this problem. By using a value for the COV greater or equal than 5 and a value for the LFC greater or equal than 2.5, a total of 42 multiclass DEGs were obtained. These genes were used as input variables for the classifiers with the main purpose of evaluating their potential as DEGs with the capability of discerning among the studied pathologies.

During the extraction process, an integration of the series from both RNA-seq and Microarray has been carried out. Thanks to this, the number of samples available for this study increased considerably. The lack of public samples from RNA-seq in comparison with Microarray was the motivation to perform the integration. Therefore, it was shown how Microarray still has a great potential in the field of gene quantification technologies and can longer be used in order to reinforce studies of this nature, increasing the number of available data and allowing to a more robust genetic signature discovery in diverse pathologies.

Moreover, thanks to the introduction of the COV parameter in the study, each DEG selected has the potential to discern among several of the 5 proposed classes. Concretely, if the maximum number of pair classes comparison are 10 and, the imposed restriction for each gene is that these DEGs discern minimum among 5, in the Table 5 it is shown how all the selected DEGs have the capability of discerning between 6 and 7 pair classes. Therefore, it makes sense to think that all the classes are discerned by any of the DEGs at any point with respect the other classes. In the validation process, theses genes have proven that they are strong candidates to be a possible gene signature that discern among the different types of leukemia studied in this article.

For the DEGs evaluation, 4 different classifiers were implemented. The results of these showed a high classification rate for the studied measures, ACC and f1-score. Furthermore, thanks to the feature selection process performed by mRMR, an optimal selection of subsets of genes for a reduced genetic signature was ensured.

Additionally, it is important to highlight that in order to ensure the correct validation of the data, a 5-fold validation has been implemented keeping an homogeneous distribution of samples of each state in each fold.

Hence, at sight of these results, with only 10 of the 42 DEGs, it is achieved a practically full discernment of the available samples under cross-validation. With only 10 genes, k-NN classifier reaches a 96.40% of ACC and a 96.28% of f1-score for the five groups considered including healthy samples. This means that the DEGs found applying our methodology, which makes use of the COV parameter, properly works to discern, with a very high precision, among the 5 proposed classes.

### Biological relevance of the DEGs

The top ten of the genes highlighted (see Fig 6) in our study were related in one way or another to peripheral blood leukocytes. Among them, DOCK2 gene which codifies by a protein involved in cytoskeleton remodelation and migration in response to chemokine signaling, has an especial relevance [54]. It has been reported that DOCK2 gene is overexpressed in chronic lymphocytic leukemia B-cells promoting their proliferation in response to Wnt5a [55]. This gene has been proposed as an drug target against leukemic cells since its expression is limited to hematopoietic tissues theoretically limiting side effects [56]. In addition, our results showed modulation of genes normally expressed in B-cells, such as BLK, BLNK and PABPC1. BLK is a proto-oncogene that encodes for a nonreceptor tyrosine-kinase involved in B-cell proliferation and differentiation. Signaling through BLK supports the pro-B to pre-B transition, growth arrest and apoptosis downstream of B-cell receptor [57]. Interestingly, this gene acts as a tumor suppressor in chronic myeloid leukemia stem cells and has been implicated in the progression of acute lymphoblastic leukemia [58, 59]. On the other hand, BLNK gene encodes for a cytoplasmic adaptor that plays a critical role in B-cell development [60]. Deficiency in this protein has been identified in some cases of pre-B acute lymphoblastic leukemia [61, 62]. In fact, the somatic loss of BLNK and concomitant mutations lead to a constitutive activation of Jak/STAT5 pathway, resulting in the generation of pre-B-cell leukemia [63]. Finally, PABPC1 encodes for a poly(A) binding protein that regulates immunoglobulin secretion in these cells [64].

Modulation of genes normally expressed in others peripheral blood leukocytes such as T-cells (LAPTM4B and EEF1A1 genes) and neutrophils (Azurocidin 1 gene) were also detected in our study. LAPTM4B gene acts downregulating the TGFB1 production in regulatory T-cells [65]. Differential expression of LAPTM4B and MIR155HG was confirmed in a small cohort of young adult NPM1-mutated cytogenetically normal acute myeloid leukemia (CN-AML) patients. Although there is no direct evidence that links LAPTM4B to leukemia, its upregulation has been shown to implicate PI3K/AKT signaling and ubiquitination pathways, both with crucial roles in leukemogenesis [66]. The gene EEF1A1 also plays a key role on the proliferation inhibition and apoptosis induction of human acute T lymphocytic leukemia cells, contributing to cancer survival in haematopoietic malignancies [67]. On the other hand, Azurocidin 1 gene encodes for a preproprotein that matures into azurophil granule antibiotic protein, with monocyte chemotactic and antimicrobial activity [68]. In chronic myeloid leukemia, this gene has been included inside a set of six genes to discriminate between tyrosine kinase inhibitor therapy responders and non-responders [69]. Dunne et al. demonstrated that downregulation of this gene correlates with a poor treatment outcome in patients with acute myeloid leukemia [70].

Finally, both RPS15 and RPS24 genes encode for ribosomal proteins that are component of the 40S subunit. Interestingly, the first one has been found in different studies to appear mutated in chronic lymphocytic leukemia patients, as it lead to impaired p53 stability [71, 72]. The second one appears mutated in Diamond-Blackfan anemia, a congenital non-regenerative hypoplastic pathology, characterized by macrocytic anemia, erythroblastopenia, and an increased risk of developing leukemia [73]. Finally, C11orf58 gene encodes for the Chromosome 11 open reading frame 58, also be known as Small Acidic Protein (SMAP) [74].

The most common mutations associated with AML are in FLT3, NPM1, CEBPA, and TP53 [75]. However, the extensive work involving the sequencing of genomes and exomes of this malignancy has revealed a variety of recurrent gene mutations associated [76]. In the same way, it is well described that ALL is a multistep disease, caused by the accumulation of mutations involving cell growth, proliferation, survival, and differentiation [77]. The introduction of genome-wide technologies has contributed to elucidate the molecular mechanisms underlying leukemic transformation in ALL and has allowed the identification of different subgroups [78].

Although the starting point of CML is well known, other genetic and cytogenetic changes play important roles in prognosis and treatment of this malignancy [79]. In this context, the mechanisms for insensitivity of CML stem remains unclear. Factors such as quiescence, high level of BCR-ABL expression, acquired mutations in the oncogene, and overexpression of membrane transporter proteins are very important [80]. CLL has the highest genetic predisposition of all hematologic neoplasms (approximately 5–10% of cases have a family history of CLL) [81]. It this disease, the genetic alterations have a great impact on the clinical course of the patients. Previous whole genome and exome sequencing studies have reveled recurrently mutated genes (such as NOTCH1, MYD88, TP53, ATM, SF3B1, FBXW7, POT1, CHD2, RPS15, IKZF3, ZNF292, ZMYM3, ARID1A, and PTPN11), but deletions of chromosome 13q14 is the most frequent aberration in CLL, occurring in 55% of cases [82].

## Conclusions

This works introduces an important evolution of the classical multiclass genomic analysis pipeline. Datasets from different technologies and from different platforms have been integrated with the purpose of collecting a higher number of samples due to the lack of RNA-seq samples of leukemia available at public databases. Moreover, this integration ensures the heterogeneity of the study. Furthermore, different types of leukemia series have been selected with the purpose of trying to find relevant biomarkers that allow to discern among the five classes. This study, as far as we are aware, was not performed yet and both, the introduced pipeline at multiclass level and the metrics for the DEGs extraction, are a very novelty step in this field.

On one hand, in this multiclass study that considers different types of leukemia, an important new parameter called COV has been used for extracting the DEGs in combination with the classical LFC. This parameter extracts biomarkers that are able to discern one or different classes from the rest using paired combinations. Moreover, the ANOVA test performed has shown that this parameter has been crucial in the development of the study. Therefore, the combination of both the LFC and the COV for multiclass biomarkers selection is an important advance in this field with very promising results.

A set of 10 DEGs have been identified as possible genetic signature by using the designed pipeline, and this set of genes has been assessed using a set of machine learning classifiers. On the other hand, the classification results at the multiclass level using the DEGs extracted has shown a high percentage for both the ACC and the f1-score metrics, overcoming the 96% with only a small subset of ten genes. At sight of these results, our DEGs can discern among the five proposed classes and can shape a powerful tool that could be very useful for the clinicians in decision making.

Thereafter, the biological study of the small subset of ten genes reveals a strong relationship between nine of the ten genes with the leukemia disease. Concretely, these genes highlighted were related with relevant biological processes such as proliferation, apoptosis or migration among others in peripheral blood leukocytes including B-cells, T-cells and neutrophils. Furthermore, these genes have been previously related to the neoplastic process in the hematopoietic tissue being especially relevant the modulation of the DOCK2 gene which has shown therapeutic implications in leukemic cells.
